# Melanocytic matricoma: Two cases of an uncommon entity

**DOI:** 10.1016/j.jdcr.2023.01.009

**Published:** 2023-01-28

**Authors:** Kathryn Haran, Michael Heaphy, Jeffrey Shackelton

**Affiliations:** a University of Nevada, Reno School of Medicine, Reno, Nevada; b Skin Cancer and Dermatology Institute, Reno School of Medicine, Reno, Nevada

**Keywords:** melanocytic matricoma, pigmented pilomatricoma

## Introduction

We report 2 cases of melanocytic matricoma, a benign pilar neoplasm. This tumor typically presents as a small, heavily pigmented papule on the head and neck of elderly men with significant sun damage. The clinical differential diagnosis includes melanoma and pigmented basal cell carcinoma. These 2 cases add to the literature by reinforcing the reproducible clinical presentation and histologic features of this uncommon neoplasm.

## Case 1

**Fig 1:**
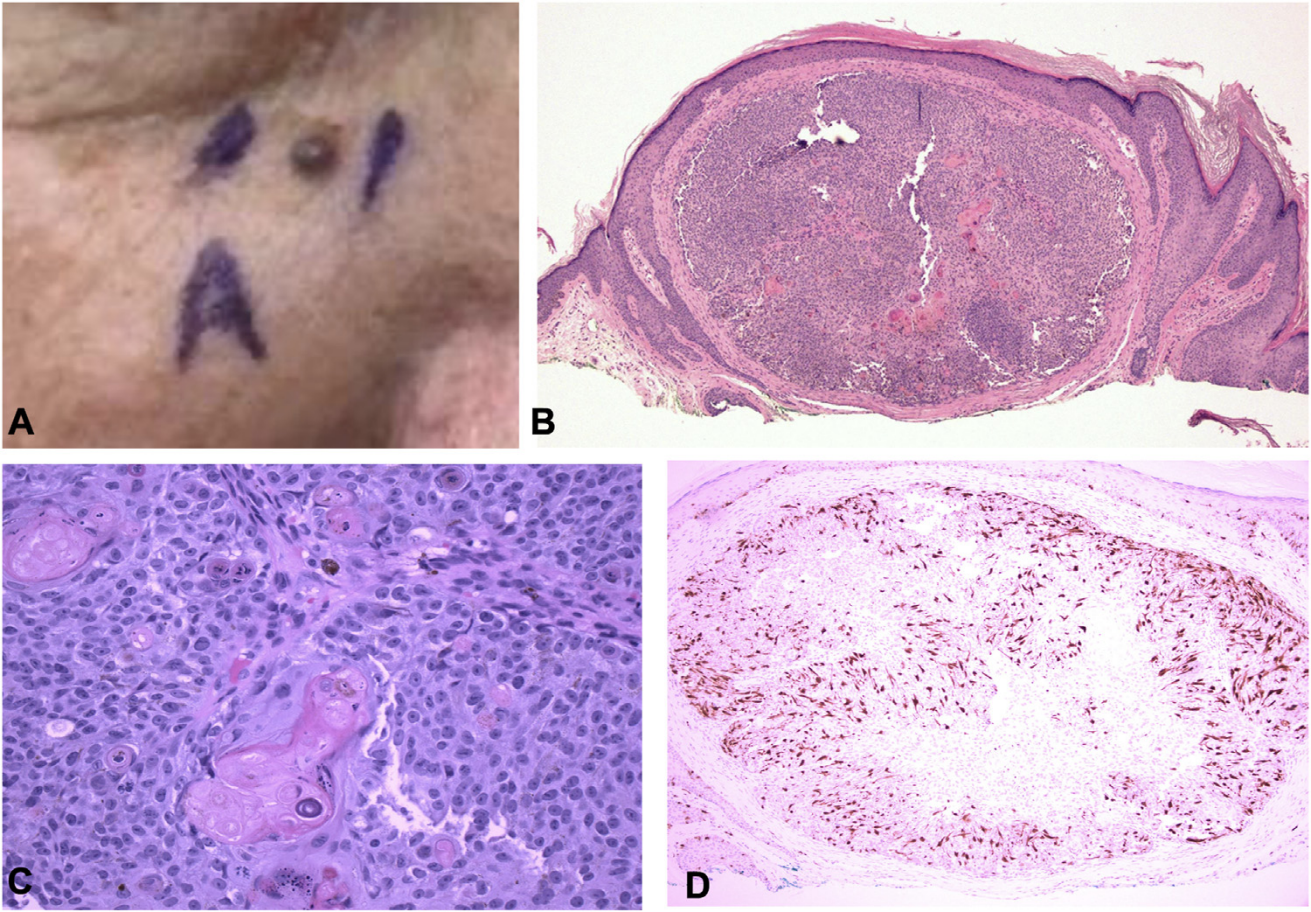
**A,** Melanocytic matricoma on the cheek of 76-year-old male with extensive sun damage. **B,** Hematoxylin and eosin stained section reveals circumscribed basaloid dermal nodule. **C,** Higher powered view reveals scattered shadow cells and melanin pigment. **D,** An S100 stained section reveals dendritic melanocytes distributed uniformly throughout the tumor.

A 76-year-old male with a history of multiple nonmelanoma skin cancers presented with a slightly pruritic black papule on his right cheek (Fig 1, *A*). The lesion had been present for 6 weeks. A shave biopsy was performed. Histologic examination revealed a well-circumscribed dermal nodule situated on heavily sun damaged skin (Fig 1, *B*). The lesion is composed of basaloid germinative cells with prominent melanin pigment, brisk mitotic activity, and scattered shadow cells (Fig 1, *C*). An S100 stained section highlights many dendritic intratumoral melanocytes (Fig 1, *D*). BerEP4 immunostaining was negative, helping to exclude pigmented nodular basal cell carcinoma.

## Case 2

**Fig 2:**
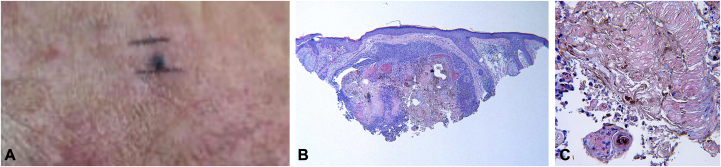
**A,** Melanocytic matricoma on the temple of 69-year-old male with extensive sun damage. **B,** Hematoxylin and eosin stained section reveals shadow cells and basaloid germinative cells with prominent melanin pigment. **C,** Prominent anucleate shadow cells.

A 69-year-old male with a history of multiple nonmelanoma skin cancers presented with an asymptomatic blue papule of unclear duration on his right temple (Fig 2, *A*). A shave biopsy was performed. Histopathology was similar to that described for case 1 (Fig 2, *B* and *C*).

## Discussion

Melanocytic matricoma was first described in 1999 by Carlson et al.^1^ Although fewer than 30 cases have been reported, the clinical and histopathologic features of this tumor are sufficiently distinctive to warrant its designation as a unique entity in the *World Health Organization Classification of Skin Tumours*.^2^

Melanocytic matricoma typically presents as a small darkly pigmented papule on sun-damaged skin of elderly male patients.^3^ Presentation on females appears to be significantly less common.^4^ The clinical differential diagnosis includes pigmented pilomatricoma, basal cell carcinoma with matrical differentiation, pigmented trichoblastoma.^1^

Histopathology typically reveals a small circumscribed dermal tumor composed of mitotically active germinative cells with differentiation toward the hair matrix. There is conspicuous melanin pigment and many dendritic melanocytes colonizing the matrical proliferation. Shadow cells are usually identified but may be scant and only present singly or in small clusters.^1^^,^^5^ The tumor is often situated in the superficial dermis with absent epidermal or adnexal connection.^4^ The matrical cells are positive for pancytokeratin, cytokeratin 5/6, and beta-catenin.^4^ The accompanying melanocytes contain thin elongated dendritic processes, are often evenly dispersed^4^ (Fig 1, *D*), and can be visualized with immunohistochemical stains for melanocytes (including Melan-A, S100, and Human Melanoma Black-45).^4^

As melanocytes normally colonize the hair matrix, it is not surprising that other matrical tumors can contain colonizing dendritic melanocytes and abundant melanin pigment, including pilomatricoma, trichoblastoma, and basal cell carcinoma with matrical differentiation. The presence or distribution of dendritic melanocytes should therefore not be used to exclude the differential diagnoses of other germinative follicular tumors.

Distinction from pigmented pilomatricoma in particular warrants discussion given the considerable overlapping features of these entities and the suggestion by some authors that these lesions could be considered on either end of a spectrum^5^ (Table I). Pigmented pilomatricoma is typically larger (often over 1 cm and often with subcutaneous extension), typically contains larger aggregates of keratin and shadow cells in a cystic configuration, and is often accompanied by foreign body granulomatous inflammation and calcification.^6^ As can be seen with the 2 cases reported here, melanocytic matricomas are typically smaller, more solid, and less cystic than pilomatricomas.^5^ The clinical setting in which these lesions are observed is also a differentiating feature—most pilomatricomas arise in children and young adults in contrast to melanocytic matricomas which typically arise in elderly males.^1^^,^^6^

**Table I:** Melanocytic matricoma versus pigmented pilomatricoma

	Clinical presentation	Size	Location	Shadow cells	Granulomatous inflammation ± calcification
Melanocytic matricoma	Elderly, particularly males, sun damaged skin	Small (diameter usually a few millimeters)	Superficial dermis	Present, may be inconspicuous	Absent
Pigmented pilomatricoma	Children and young adults	Large (diameter often over 1 cm)	Pandermal, often with subcutaneous extension	Present in large aggregates	Present

Melanocytic matricoma is often readily distinguished from pigmented trichoblastoma by the presence of a follicular stromal component and absent shadow cells in the latter.^7^ Finally, basal cell carcinoma with matrical differentiation should demonstrate typical features of basal cell carcinoma, including peripheral palisading of tumor cells and stromal retraction.^8^ In unusual cases, BerEP4 immunopositivity would favor basal cell carcinoma over melanocytic matricoma.^4^

In summary, these 2 cases demonstrate the unique clinical and histologic features of melanocytic matricoma, an entity that can clinically mimic melanoma and histologically mimic other basaloid germinative tumors. These cases contribute to the literature as they reinforce the clinical presentation of melanocytic matricoma, with both cases presenting as small, pigmented papules on sun damaged skin of elderly men.^3^

## Conflicts of interest

None disclosed.
